# Supplementary material for: Vitamin B5 metabolism is essential for vacuolar and mitochondrial functions and drug detoxification in fungi
Source: Commun Biol. 2024 Jul 23;7:894. doi: 10.1038/s42003-024-06595-7 (PMC11266677; doi:10.1038/s42003-024-06595-7)
Supplement: Supplementary file 2 — Supplementary Material [file 42003_2024_6595_MOESM2_ESM.pdf]

Vitamin B5 Metabolism is Essential for Vacuolar and Mitochondrial Functions and Drug Detoxification in Fungi

Jae-Yeon Choi, Shalev Gihaz, Muhammad Munshi, Pallavi Singh, Pratap Vydyam, Patrice Hamel, Emily M. Adams, Xinghui Sun, Oleh Khalimonchuk, Kevin Fuller, and Choukri Ben Mamoun#

#Corresponding author:
Choukri Ben Mamoun: choukri.benmamoun@yale.edu

This PDF file includes:

Fig. S1 to S8

Tables S1

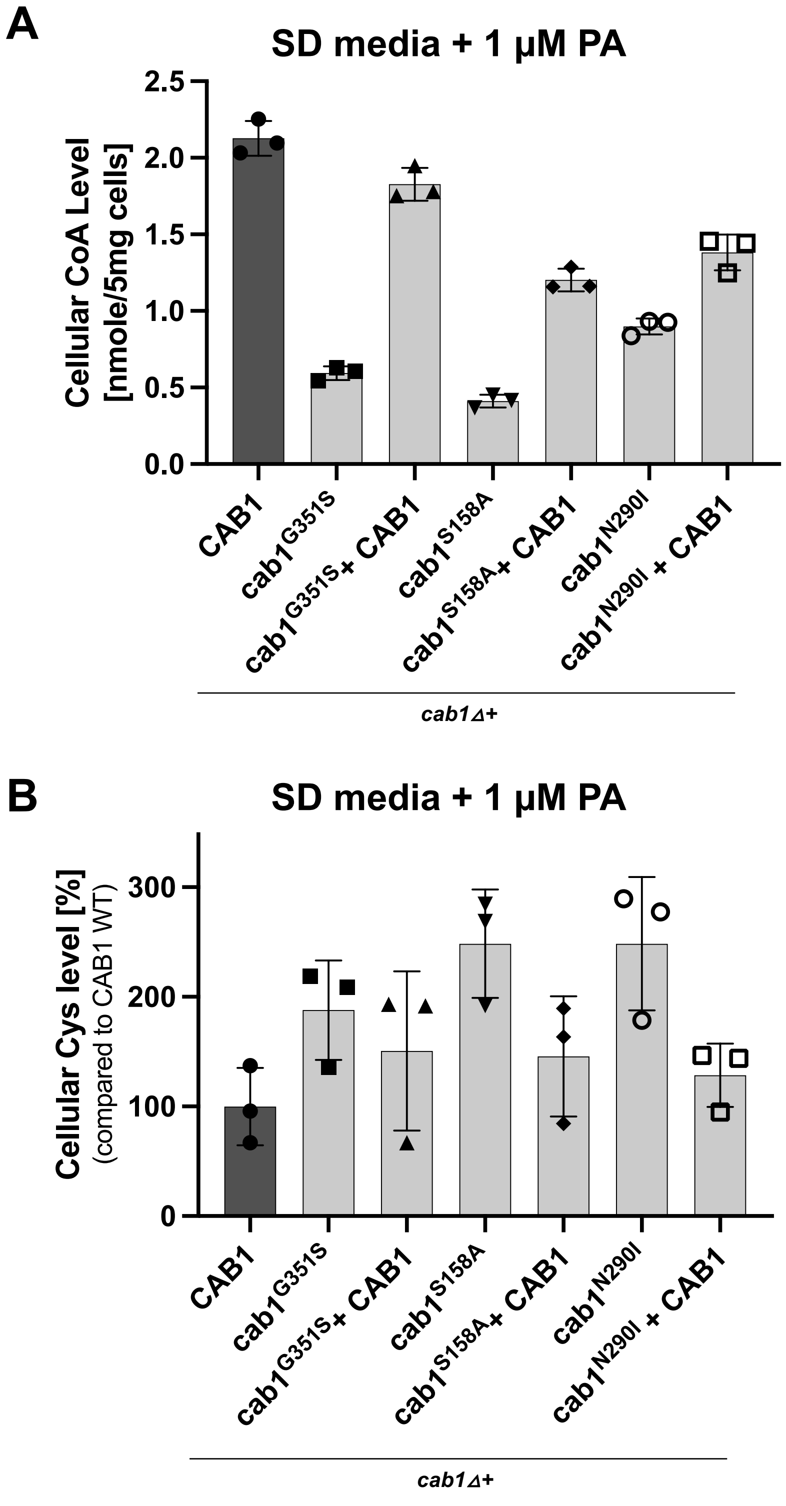

Fig. S1. Cellular CoA (A) and cysteine (B) levels in *cab1*Δ strains harboring various *CAB1* mutations. CoA and cysteine levels were measured using the metabolite extracts from the yeast strains grown in the presence of 1 μM PA. The data represents average of three independent experiments (±SD).

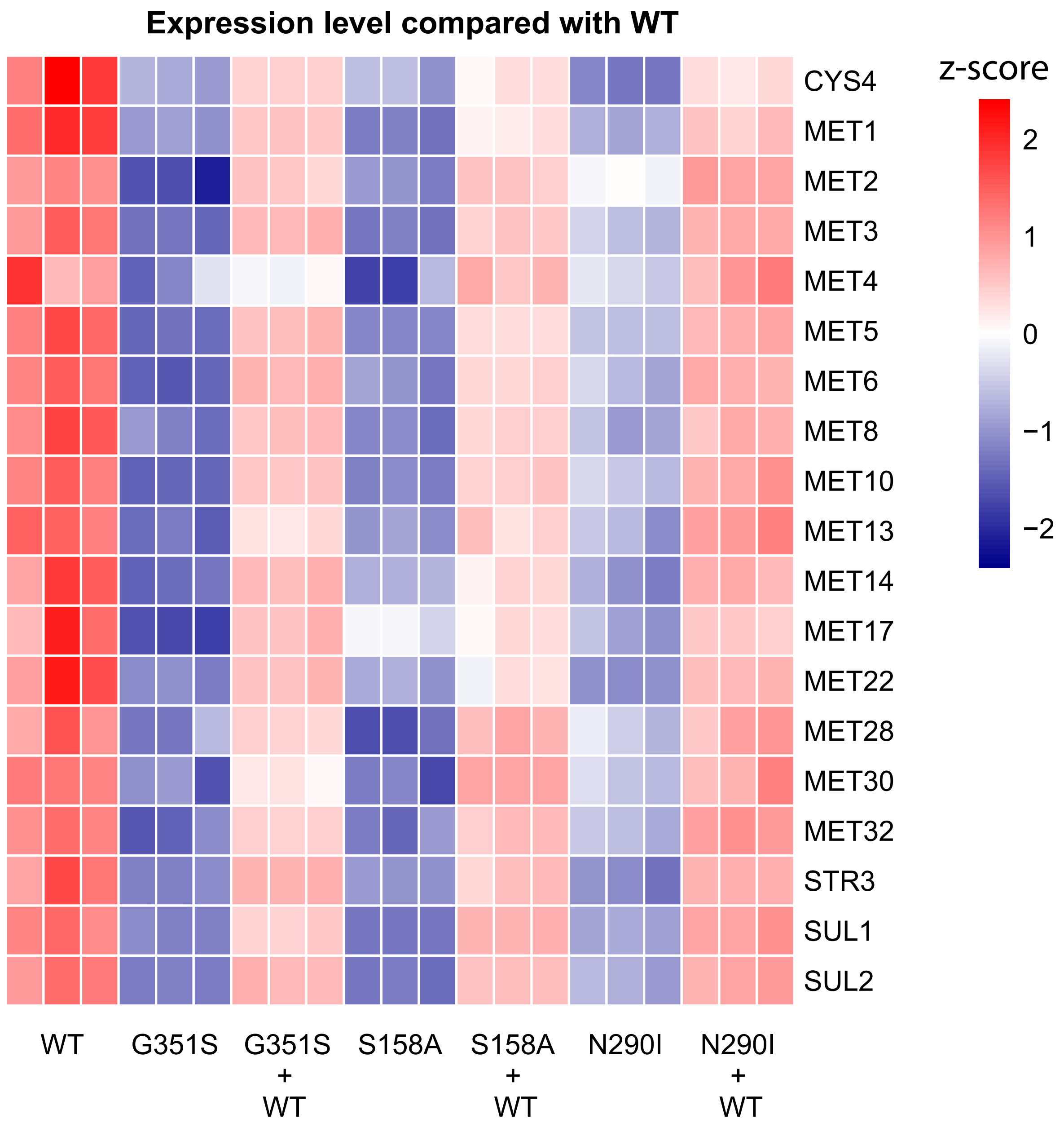

Fig. S2. RNA-Seq analysis for cysteine and sulfur homeostasis genes expressed in *cab1*Δ strains. The results are based on normalized TMM compared with the expression profile of the WT parent strain. The gene list with annotations shown in Table S1. The data represent the average of three biological samples.

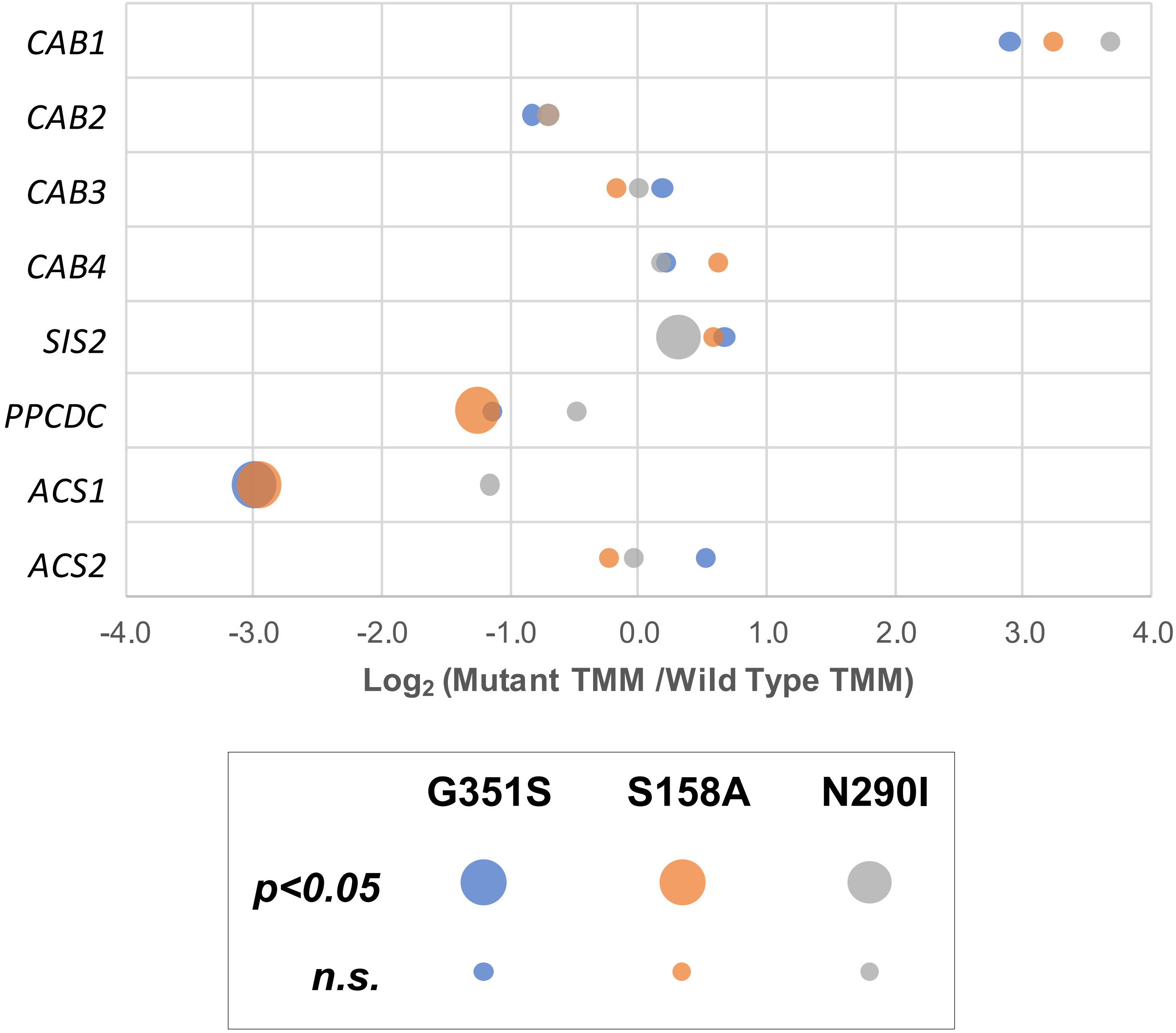

Fig. S3. RNA-Seq analysis for PCA pathway genes expressed in *cab1*Δ strains harboring various *CAB1* mutations. Large circles in corresponds to genes for which the p-value (WT vs. mutant) <0.05, but the p-value (WT vs. addback) >0.05, indicating the null hypothesis of equal expression was rejected for the mutant, but not rejected for the addback at p=0.05. Small circles correspond to genes for which either of these criteria were not met. The full gene list is shown in Supplementary Table 1. The data represent the average of three biological samples.

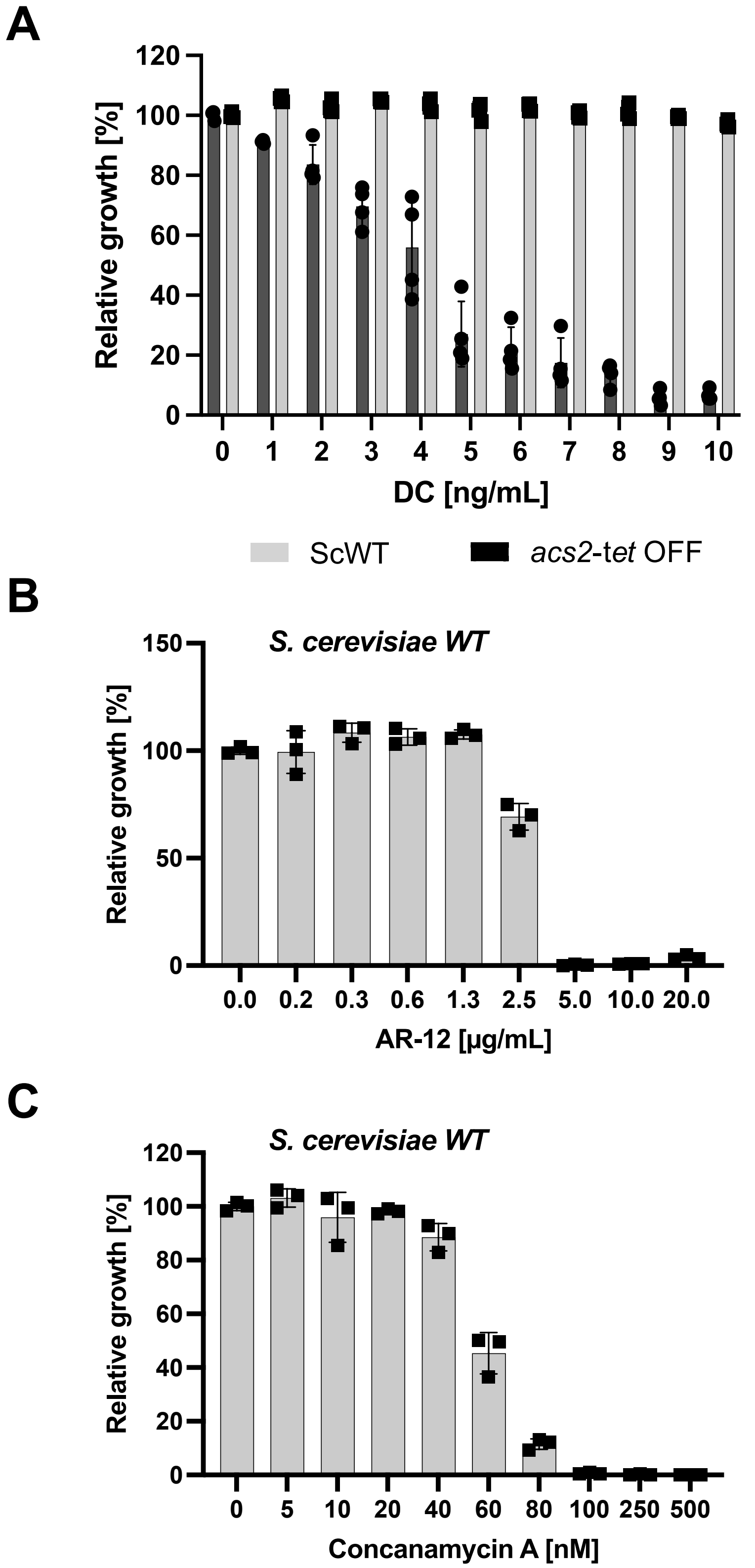

Fig. S4. Effect of modulation of downstream steps from the PCA pathway on the growth of *S. cerevisiae*. *S. cerevisiae* (WT or acs2-tetoff mutant, as mentioned) cells were inoculated in the presence or absence of rising concentration of A) doxycycline, B) AR-12, and C) concanamycin A, at 30°C for 24-48 h. The growth was normalized to DMSO treated wells (no drug=100% growth) and 200 µM amorolfine well (0% growth). The data represent the average of 4 biological samples (±SD).

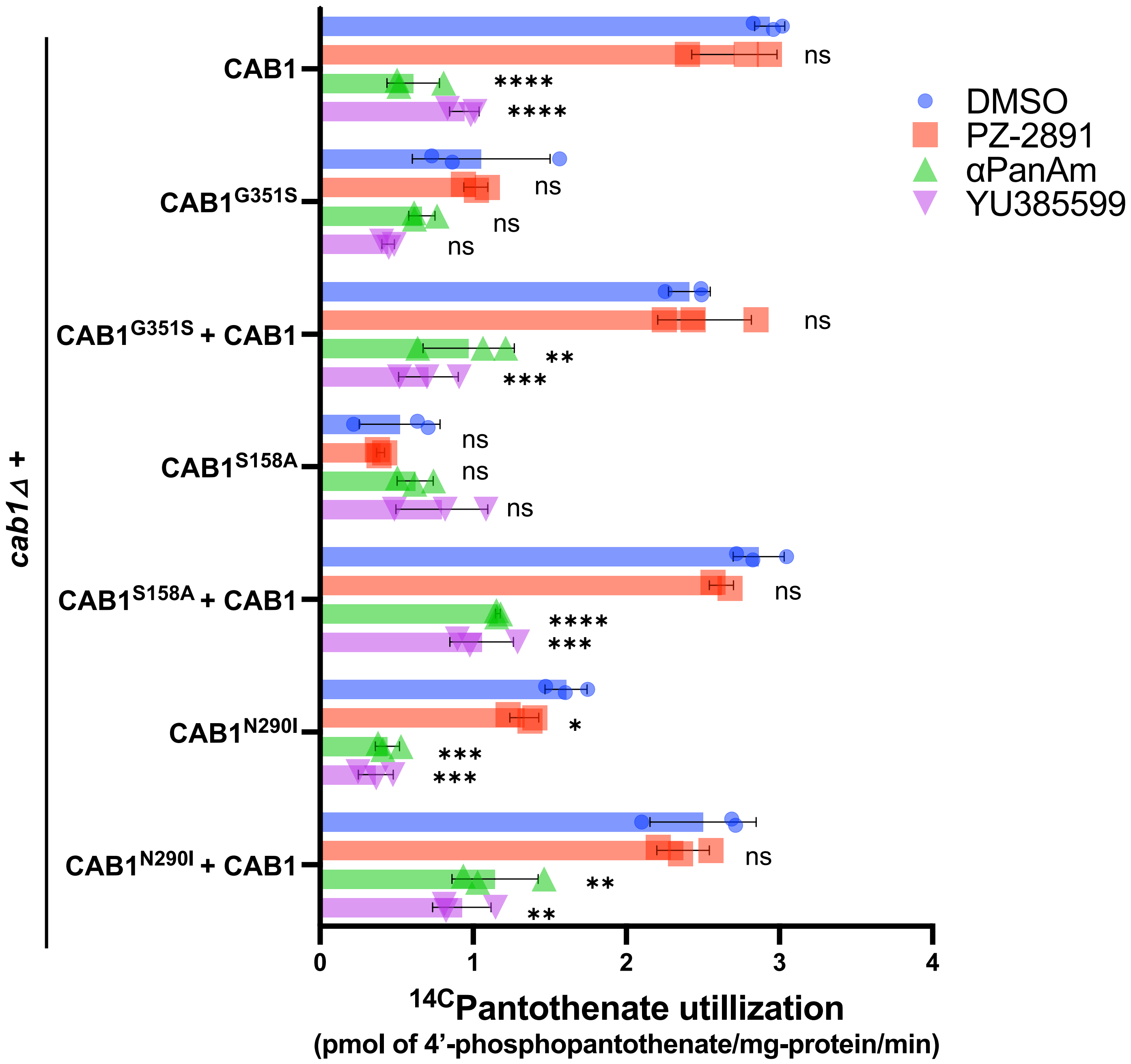

Fig. S5. Pantothenate Kinase activity in the cab1Δ strains harboring various CAB1 mutations. Cell free extracts of yeast expressing cab1 mutants were used to measure the endogenous Pank activity of cab1 variants using D-[1-14C] pantothenate as a substrate for 10 min at 30°C. The Pank activity was measured in the absence or presence of 20 μM PZ-2891 (hPanK3 activator), α-PanAm (known Cab1 inhibitor), and YU385599 (reported Cab1 inhibitor). The data represent an average of three independent experiments (±SD). Statistical significance was determined using t-test ($p=0.05$) with GraphPad Prism and the corresponding p-values (****$p < 0.0001$, ***$p < 0.001$, **$p < 0.01$, *$p < 0.05$, and ns > 0.05) are indicated.

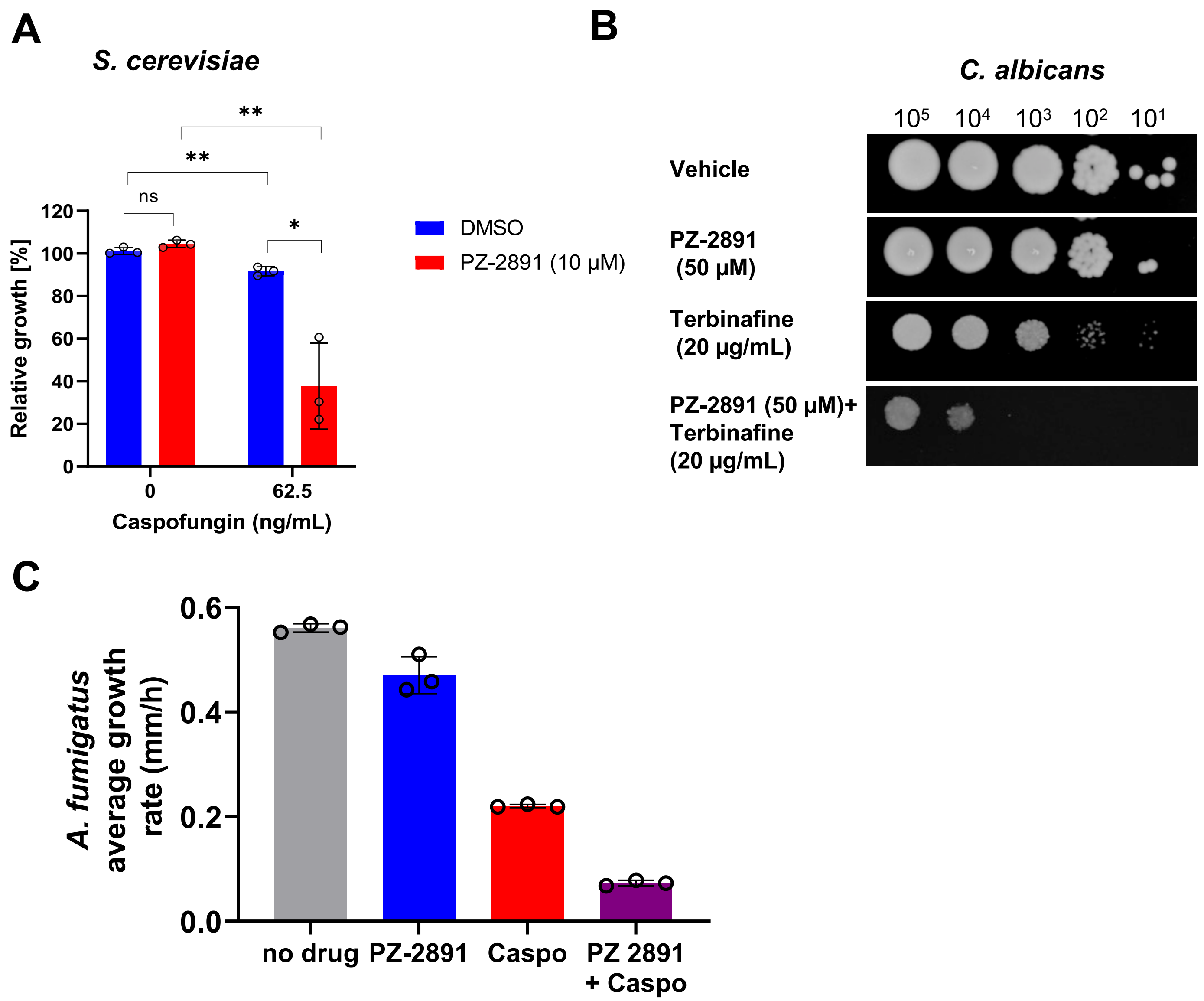

Fig. S6. Potentiation of PZ-2891 on antifungal susceptibility in different yeast species. A) caspofungin efficacy in S. cerevisiae with PZ-2891. S. cerevisiae cells were inoculated in the presence or absence PZ-2891, in combination with caspofungin treatment at 30°C for 24-48 h. The growth was normalized to DMSO treated wells (no drug=100% growth) and 200 μM amorolfine well (0% growth). For these assays, t-test was done among the mentioned groups (p=0.05). Liquid growth assays were conducted in quadruplicate (n = 4) and the plotted graphs represent the average of 4 data sets ± SD. Statistical significance was determined using t-test (p=0.05) with GraphPad Prism and the corresponding p-values (**p < 0.01, *p < 0.05, and ns > 0.05) are indicated. B) Potentiation of terbinafine efficacy in C. albicans with PZ-2891. C. albicans spotting growth assays were performed when cells were inoculated into YPD overnight, harvested, washed, and re-suspended in 0.9% NaCl. Serial dilutions of cells were spotted onto YPD plates containing terbinafine (20 μg/mL) in the presence or absence of PZ-2891 (50 μM) at 30°C for 4 days. The representative images are from two independent experiments, each performed in duplicates. C) Average growth rate (based on colony diameter) of A. fumigatus colonies in the presence or absence of PZ-2891 (50 μM) in combination with caspofungin treatment (20 μg/ml). The results were calculated after 72 h of growth. The data represent average of 3 independent experiments ± SD.

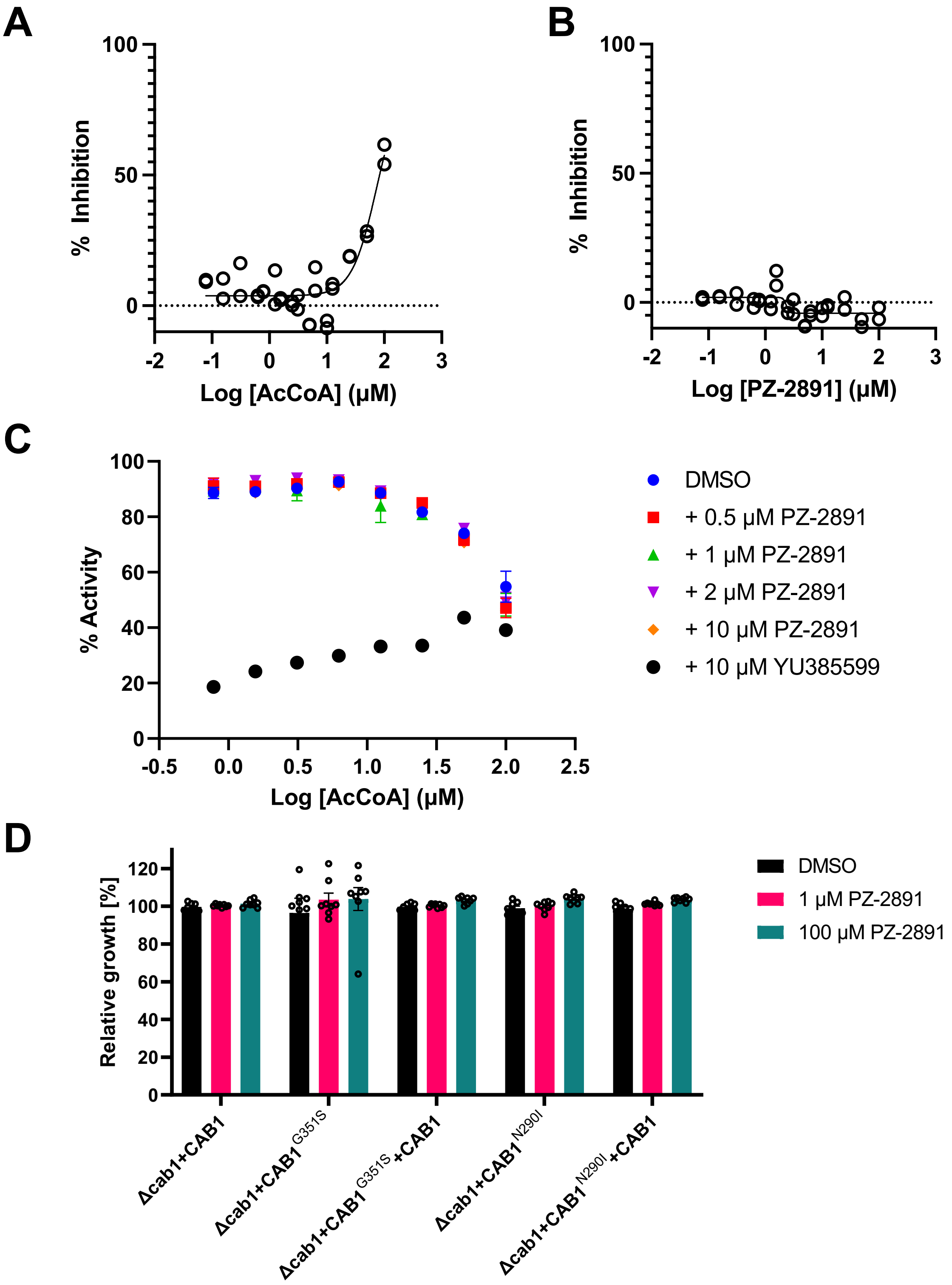

Fig. S7. PZ-2891 does not have inhibitory effect on either S. cerevisiae Cab1 enzymatic activity or *S. cerevisiae* cell growth. A) Dose-response curve for AcCoA effect on recombinant Cab1 enzyme activity. B) Dose-response curve for PZ-2891 effect on recombinant Cab1 enzyme activity. C) Dose-response curve for AcCoA effect on recombinant Cab1 enzyme activity in the absence of presence of PZ-2891 or YU385599 (inhibitor control). The data (A to C) represent average of 3 independent experiments ± SD. D) Liquid growth assay for cab1Δ strains harboring various *CAB1* mutations in the presence or absence of 1-100 μM PZ-2891. Liquid growth assays were conducted in quadruplicate (n = 4) and the plotted graphs represent the average of 4 data sets ± SD.

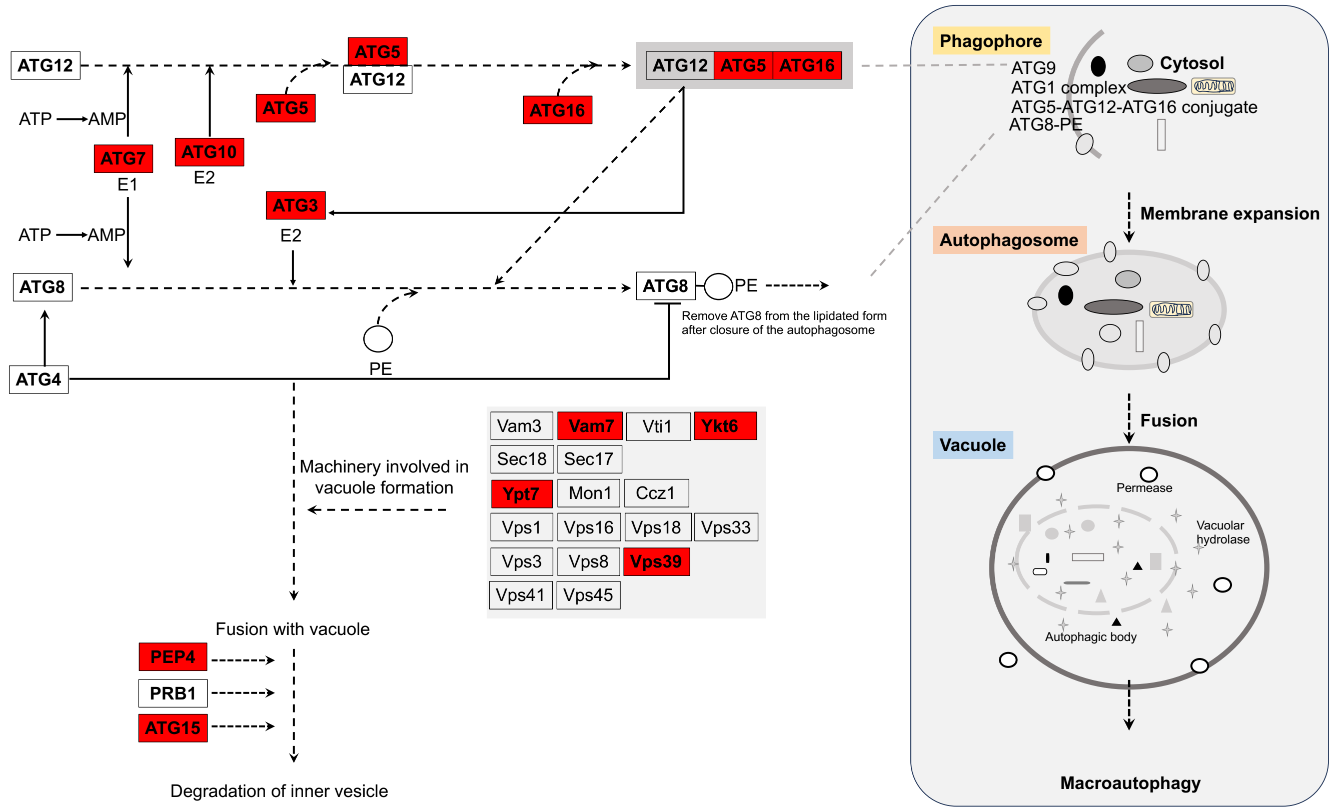

Fig. S8. Genes involved in autophagy display negative genetic interactions with the *CAB1* mutation. Genes involved in negative genetic interactions with a *cab1* mutant, particularly those involved in the autophagy pathway, were identified using YeastMine and ShinyGO tools, as detailed in the methods section. The pathway was assembled from pre-drawn KEGG pathways and further refined using PowerPoint program to emphasize specific interactions and functional annotations pertinent to autophagosome formation, as well as the subsequent processes of vacuolar fusion and degradation of inner vesicle. All genes encoding the proteins in the depicted pathway are shown, with the specific genes interacting with a cab1 mutant highlighted in red.

Table S1. Down regulated genes list associated with *CAB1* deficiency

Systematic name	Standard name and aliases	Short description
YDR531W	CAB1, pantothenate kinase	Pantothenate kinase, ATP:D-pantothenate 4'-phosphotransferase
YIL083C	CAB2, phosphopantothenate--cysteine ligase CAB2	Phosphopantothenoylcysteine synthetase (PPCS)
YKL088W	CAB3, phosphopantothenoylcysteine decarboxylase complex subunit CAB3	Subunit of PPCDC and CoA-SPC complexes involved in CoA biosynthesis
YGR277C	CAB4, putative pantetheine-phosphate adenylyltransferase	Subunit of the CoA-Synthesizing Protein Complex (CoA-SPC)
YKR072C	SIS2,HAL3,phosphopantothenoylcysteine decarboxylase complex subunit SIS2	Negative regulatory subunit of protein phosphatase 1 (Ppz1p)
YOR054C	VHS3,YOR29-05,phosphopantothenoylcysteine decarboxylase complex subunit VHS3	Negative regulatory subunit of protein phosphatase 1 Ppz1p
YAL054C	ACS1, FUN44,acetate--CoA ligase 1	Acetyl-coA synthetase isoform
YLR153C	ACS2, acetate--CoA ligase ACS2	Acetyl-coA synthetase isoform
YBR294W	SUL1,SFP2,sulfate permease	High affinity sulfate permease of the SulP anion transporter family
YLR092W	SUL2,sulfate permease	High affinity sulfate permease
YKR069W	MET1, MET20,uroporphyrinogen-III C-methyltransferase	S-adenosyl-L-methionine uroporphyrinogen III transmethylase
YNL277W	MET2, homoserine O-acetyltransferase	L-homoserine-O-acetyltransferase
YJR010W	MET3, sulfate adenylyltransferase	ATP sulfurylase
YJR137C	MET5,ECM17,sulfite reductase (NADPH) subunit beta	Sulfite reductase beta subunit
YER091C	MET6,5-methyltetrahydropteroyltriglutamate-homocysteine S-methyltransferase	Cobalamin-independent methionine synthase
YBR213W	MET8, bifunctional precorrin-2 dehydrogenase/sirohydrochlorin ferrochelatase MET8	Bifunctional dehydrogenase and ferrochelatase
YFR030W	MET10,sulfite reductase subunit alpha	Subunit alpha of assimilatory sulfite reductase
YGL125W	MET13,MET11,MRPL45,methylenetetrahydrofolate reductase (NAD(P)H) MET13	Major isozyme of methylenetetrahydrofolate reductase
YKL001C	MET14, adenylyl-sulfate kinase	Adenylylsulfate kinase
YLR303W	MET1, MET15,MET25,bifunctional cysteine synthase/O acetylhomoserine aminocarboxypropyltransferase MET17	O-acetyl homoserine-O-acetyl serine sulfhydrylase
YOL064C	MET22,3'(2'),5'-bisphosphate nucleotidase,HAL2	Bisphosphate-3'-nucleotidase
YIR017C	MET28	bZIP transcriptional activator in the Cbf1p-Met4p-Met28p complex
YDR253C	MET32	Zinc-finger DNA-binding transcription factor
YGL184C	STR3,cystathionine beta-lyase STR3	Peroxisomal cystathionine beta-lyase
YGR155W	CYS4,NHS5,STR4,VMA41,cystathionine beta-synthase CYS4	Cystathionine beta-synthase
