# Supplementary material for: Vitamin B5 metabolism is essential for vacuolar and mitochondrial functions and drug detoxification in fungi
Source: Commun Biol. 2024 Jul 23;7:894. doi: 10.1038/s42003-024-06595-7 (PMC11266677; doi:10.1038/s42003-024-06595-7)
Supplement: Supplementary file 3 — Description of Additional Supplementary Files [file 42003_2024_6595_MOESM3_ESM.pdf]

Description of Additional Supplementary Files

File name: Supplementary Data
Description: Source data underlying the graphs and charts presented in the main figures.
